# *WDR4* gene polymorphisms increase hepatoblastoma susceptibility in girls

**DOI:** 10.7150/jca.76255

**Published:** 2022-09-21

**Authors:** Shaohua He, Jinhong Zhu, Zhixiang Xiao, Jiabin Liu, Jiao Zhang, Yong Li, Zhonghua Yang, Jiwen Cheng, Suhong Li, Li Li, Jing He, Di Xu

**Affiliations:** 1 Department of Pediatric Surgery, Shengli Clinical Medical College of Fujian Medical University, Fuzhou 350001, Fujian, China.; 2 Department of Clinical Laboratory, Biobank, Harbin Medical University Cancer Hospital, Harbin 150040, Heilongjiang, China.; 3 Department of Pediatric Surgery, Guangzhou Institute of Pediatrics, Guangdong Provincial Key Laboratory of Research in Structural Birth Defect Disease, Guangzhou Women and Children's Medical Center, Guangzhou Medical University, Guangzhou 510623, Guangdong, China.; 4 Department of Pediatric Surgery, the First Affiliated Hospital of Zhengzhou University, Zhengzhou 450052, Henan, China.; 5 Department of Pediatric Surgery, Hunan Children's Hospital, Changsha 410004, Hunan, China.; 6 Department of Pediatric Surgery, Shengjing Hospital of China Medical University, Shenyang 110004, Liaoning, China.; 7 Department of Pediatric Surgery, the Second Affiliated Hospital of Xi'an Jiaotong University, Xi'an 710004, Shaanxi, China.; 8 Department of Pathology, Children Hospital and Women Health Center of Shanxi, Taiyuan 030013, Shannxi, China.; 9 Kunming Key Laboratory of Children Infection and Immunity, Yunnan Key Laboratory of Children's Major Disease Research, Yunnan Institute of Pediatrics Research, Yunnan Medical Center for Pediatric Diseases, Kunming Children's Hospital, Kunming 650228, Yunnan, China.

**Keywords:** hepatoblastoma, susceptibility, *WDR4*, polymorphism, m7G

## Abstract

Hepatoblastoma, originating from hepatoblasts, is the most common hepatic malignancy. WD repeat domain 4 (WDR4) is a subunit of RNA N(7)-methylguanine (m7G) methyltransferase complex. Recently, WDR4 has shown oncogenic potential in various adult cancers, but its roles in pediatric cancers have not been reported. We performed a case-control study (313 cases vs. 1446 controls) to investigate whether genetic variants in the *WDR4* gene influence hepatoblastoma susceptibility in the Chinese Han nationality. We first determine the genotypes of five *WDR4* gene polymorphisms (rs2156315 C>T, rs2156316 C>G, rs6586250 C>T, rs15736 G>A, rs2248490 C>G) in participants, using the Taqman assay. And then, an unconditional logistic regression analysis was performed to assess the association between *WDR4* gene polymorphisms and hepatoblastoma risk. Overall, we did not find any polymorphism significantly associated with the risk of developing hepatoblastoma. Instead, the stratified analysis revealed that the co-existence of 2-5 risk genotypes increased hepatoblastoma risk by 2.23 folds in girls (adjusted odds ratio=2.23, 95% confidence interval=1.17-4.23, *P*=0.014). In conclusion, our results demonstrate that single selected polymorphisms were too weak to exert a significant effect on the whole study population. However, in combination, two or more *WDR4* gene polymorphisms significantly conferred increased hepatoblastoma risk in girls. Our findings may encourage more genetic association studies to discover significant polymorphisms in the *WDR4* gene for hepatoblastoma.

## Introduction

Hepatoblastoma is an embryonal tumor arising from hepatoblast, a hepatocyte precursor cell, characterized by heterogeneous histological patterns with the presence of different stages of liver development cancer 1. Even though the annual incidence is as low as 1.5 cases per million, hepatoblastoma is the most common hepatic malignancy in childhood. Most hepatoblastoma-affected children are below the age of 3 years, and the median age at the time of diagnosis is one year.

Epidemiology studies suggest prematurity, low birth weight, and parental smoking may be the risk for hepatoblastoma 2. Apart from developmental processes and environmental factors, genetic predisposition is also a major factor influencing the risk of childhood cancer 3. Several inherited syndromes have predisposed children to hepatoblastoma, including Familial Adenomatous Polyposis (FAP) with inactivating mutations of the *APC* gene, Beckwith-Wiedemann syndrome (BWS) with defective imprinting of the *IGF2*-*H19* locus on chromosome 11p15, and Trisomy 18 (Edwards Syndrome) 2. Besides the major predisposition syndromes, more subtle genetic factors are also involved in hepatoblastoma susceptibility. The Nagae group integratively and comprehensively analyzed the genetic and epigenetic landscape of hepatoblastoma in a Japanese cohort. They revealed telomerase reverse transcriptase (TERT) promoter mutations and the specific hypomethylated enhancers in the transcription factor ASCL2 targeted genes in hepatoblastoma 4. Moreover, susceptibility loci (e.g., genetic polymorphisms) can be inherited, leading to an increased risk of diseases in descendants. Several teams and ours have shown that hereditary single nucleotide polymorphisms (SNPs) in a number of genes can alter hepatoblastoma susceptibility 5-11. Due to the disease's rarity, molecular epidemiological studies on hepatoblastoma are extraordinarily few and lagged behind adult cancers. Therefore, it is imperative to identify more hepatoblastoma predisposing genes with case-control studies.

The human chromosome 21q22.3 gene *WD repeat domain 4* (*WDR4*) encodes a subunit of a methyltransferase complex required for the formation of N(7)-methylguanine (m7G) 12, 13. WDR4 complexes with and facilitates METTL1, the catalytic component, to install m7G in the cap structure of mRNA and specific sites internally within tRNA, rRNA, miRNA, and mRNA 14-19. Accumulating evidence has suggested *WDR4* as an oncogenic gene 14, 20-23. Pan-cancer analysis of 33 types of cancer indicated WDR4 was aberrantly upregulated in various cancers 20. Recently, two critical studies demonstrated that the METTL1/WDR4 complex increased oncogenic mRNA translation and drove malignant transformation by facilitating m7G modification of a subset of tRNAs 21, 22. The implications of WDR4 in hepatoblastoma have not been reported, but it was reported to facilitate proliferation, metastasis, and sorafenib resistance in hepatocellular carcinoma 23. Because of its tumorigenic role, we analyzed five *WDR4* gene polymorphisms in 313 pediatric patients and 1446 controls to explore whether functional *WDR4* gene variants can modify individuals' cancer susceptibility.

## Materials and Materials

### Study population

The pediatric cohort consisted of 313 children with hepatoblastoma collected from seven independent hospitals across China. All patients were children with newly diagnosed hepatoblastoma, which two or more pathologists confirmed. Hepatoblastoma patients were recruited based on the following inclusion and exclusion criteria: 1) Han Chinese descendants, 2) newly diagnosed and histopathologically validated hepatoblastoma, 3) lack of familial disorder or family history of cancer, and 4) ≤14 years old. Patients receiving the medical intervention or failing to provide signed informed consent would be excluded. The locations of these hospitals were as follows: Guangzhou, Kunming, Xi'an, Zhengzhou, Changsha, Taiyuan, and Shenyang. We also matched cases with 1446 healthy controls of Chinese Han nationality, who were children visiting the above hospitals for physical examination at the same periods as cases (**Table S1**). The clinical stages of the patients were determined following the PRETEXT classification 24. Information on subject recruitments and participants' epidemiological and clinical characteristics were provided elsewhere 9, 11. Signed informed consent by parents or guardians of each subject was a prerequisite for blood withdrawal. The study protocol was approved by the institutional review board of Guangzhou Women and Children's Medical Center (No: 202016601).

### Selection and genotyping of SNPs

Based on standard criteria 25, 26, we selected five potential functional SNPs (rs2156315 C>T, rs2156316 C>G, rs6586250 C>T, rs15736 G>A, and rs2248490 C>G) in the *WRD4* gene. Briefly, we obtained all *WDR4* gene SNPs from the dbSNP database (http://www.ncbi.nlm.nih.gov/projects/SNP) and then selected common, potentially functional SNPs SNPinfo software (https://snpinfo.niehs.nih.gov/snpinfo/snpfunc.html). These SNPs also are in low linkage disequilibrium (LD) (R^2^<0.8) with one another and have a minor allele frequency (MAF) equal to or larger than 5% in the Chinese Han population. The Tiangen Blood DNA Extraction kits (Tiangen Biotechnology, Beijing, China) were adopted to collect the Genomic DNA of all children, followed by genotyping using a TaqMan platform (Applied Biosystems, Foster City, CA). We routinely added both negative and positive control samples to each 384-well plate. Blinded laboratory workers finished all the experiments. Quality control was executed by regularly genotyping a certain fraction of the randomly selected sample. Concordance rates of 100% in duplicate tests were accepted.

### Statistical analysis

To determine the significant differences in clinical variables between the case and control groups, t-tests and χ^2^ tests were performed for continuous or categorical variables, respectively. Hardy-Weinberg equilibrium (HWE) was checked in the control subjects using a goodness-of-fit χ^2^ test. The association of SNPs and hepatoblastoma susceptibility was estimated using unconditional logistic regression analysis, along with the generation odds ratios (ORs) and 95% confidence intervals (CIs). Children were also stratified regarding age, sex, and clinical stages, and the associations were reanalyzed in subgroups. The nominal significance level α was set at 0.05. All statistical analyses were carried out by SAS v9.1 (SAS Institute Inc., Cary, NC).

## Results

### Association of hepatoblastoma risk with *WDR4* SNPs

We investigated five *WDR4* gene SNPs in this study, including rs2156315 C>T, rs2156316 C>G, rs6586250 C>T, rs15736 G>A, and rs2248490 C>G. Out of the 313 hepatoblastoma cases and 1446 controls, the SNPs were successfully genotyped in 308 cases and 1444 controls. All genotyping results for hepatoblastoma patients and controls are displayed in **Table 1**. We confirmed the consistency with HWE genetic balance in control subjects for the five SNPs (HWE=0.063 for rs2156315 C>T, HWE=0.459 for rs2156316 C>G, HWE=0.897 for rs6586250 C>T, HWE=0.966 for rs15736 G>A, HWE=0.387 for rs2248490 C>G). However, neither single-locus nor combined analysis detected a significant association between the five SNPs and hepatoblastoma risk, suggesting that the effects of these SNPs on susceptibility are very weak.

### Stratified analyses

We next performed stratified analysis for rs2156315 because it has the largest OR value of 1.42, although not significant (**Table 2**). However, this SNP did not show significant association in any subgroups in terms of age, gender, and stages. Regardless of significance, we defined genotypes with OR of >1 as risk genotype: rs2156315 TT, rs2156316 GG, rs6586250 TT, rs15736 AA, and rs2248490 CC/CG. The combined analysis of the five risk genotypes showed that 2-5 risk genotypes carriers were more prevalent in girls than in boys (30% vs. 19%). When compared with girls carrying 0-1 risk genotypes, girl carriers of 2-5 risk genotypes were associated with a 2.23-fold increased risk of hepatoblastoma (adjusted OR=2.23, 95% CI=1.17-4.23, *P*=0.014).

## Discussion

Despite the extremely low incidence of hepatoblastoma, early diagnosis is a critical factor in achieving a high cure rate. Numerous studies have shown that small risk factors like genetic polymorphisms can modify cancer risk in conjunction with adverse environmental factors. It is also the case of hepatoblastoma 3, 27, 28. However, the rarity of hepatoblastoma constitutes a severe obstacle to launching large case-control studies. As a result, case-control studies on hepatoblastoma are very few, and sample sizes are small. For example, Pakakasama et al. elucidated a significant association between *MPO* gene promoter SNP (G to A) and reduced risk of hepatoblastoma, but they only recruited 48 cases and 180 healthy controls of Caucasians 5. Again, with only 84 children with hepatoblastoma, the same team studied found that a nonsynonymous SNP located (G to A) at codon 242 of *CCND1* could significantly affect the age of the tumor onset 5. Therefore, much more work is warranted to elucidate the significance of numerous genetic polymorphisms in hepatoblastoma risk with large case-control studies.

After have being engaged in genetic association studies in pediatric cancers for over a decade, our team has found a number of hepatoblastoma susceptibility genes with 313 cases and 1446 controls, including *LINC00673* 29, *NRAS* 30, *KRAS* 30, *TP53* 31, *HMGA2* 32, *miR-34b/c* 31, as well as DNA repair genes 6, 7. Moreover, we also identified genetic variants in RNA m6A-mediated genes (i.e., *METTL3* 33, *METTL14* 34, *WTAP* 9, *YTHDF1* 11, *YTHDC1* 10, and *ALKBH5* 35) associated with hepatoblastoma susceptibility. Over the past years, research gradually realized that, like RNA m6A modification, the abundant RNA m7G modifications also have fundamental importance to cellular homeostasis. However, molecular epidemiological studies of s *WDR4* genetic variants and disease risk are still in infancy. There is a substantial gap in our knowledge of the relationship between *WDR4* gene variants and disease susceptibility. Only one study suggests that *WDR4* gene variants may be disease-causing. Wang et al. demonstrated that the *WDR4* gene rs465663 polymorphism was associated with asthenozoospermia. Functional annotations using the GTEx portal indicated that the TT or TC genotype of SNP rs465663 was associated with decreased expression of *WDR4* compared with the CC genotype. Furthermore, the functional study elucidated that mwdr4 heterozygous (+/-) mouse embryonic fibroblasts were more vulnerable to ROS-induced DNA fragmentation than wild-type counterparts 36. In the current study, we analyzed five potential functional *WDR4* gene SNPs with 313 cases and 1446 controls, and found that Chinese girls bearing two or more risk genotypes were more likely to develop hepatoblastoma than girls with few risk genotypes. Our results provided evidence that *WDR4* may be a hepatoblastoma susceptibility gene.

WDR4 is a non-catalytic component of the METTL1/WDR4 methyltransferase complex, essential for the stability and conformational alterations of METTL1, the catalytic subunit 12. METTL1/WDR4 complex-medicated m7G modification is indispensable in controlling cell fate and growth, and WDR4 has been implicated in carcinogenesis. A missense mutation in *WDR4*, impairing tRNA m (7) G46 methylation, was identified as a causal variant for microcephalic primordial dwarfism 37. Knockout of METT1 or WDR4 was detrimental to self-renewal and neural differentiation of mouse embryonic stem cells 16. Pan-cancer analysis based on the TCGA database revealed aberrant expression of WDR4 in a broad spectrum of cancers and the association with inferior prognosis as well as tumor immunity 20. WDR4 coupled with METTL1 facilitates the mounting of m7G modification at position 46 in tRNAs. Ma's group found that both WDR4 and METTL1 were significantly upregulated in human lung cancer. *In vitro* and *in vivo* evidence indicated that defective m7G tRNA modification resulting from the knockdown of WDR4/METTL1 suppressed cell proliferation, invasion, and tumorigenic capacity, while overexpression of these two methyltransferases promoted tumorigenesis 15. Xia and colleagues substantiated the upregulated WDR4 in hepatocellular carcinoma (HCC), accompanied by increased m7G methylation levels. Overexpressed WDR4 boosted proliferation and the G2/M cell cycle progression but inhibited apoptosis of HCC cells by enhancing mRNA stability and translation of CCNB1, which encodes the cell cycle protein cyclin B1 23. Moreover, elevated METTL1/WDR4 and m7G tRNA modification were also observed in intrahepatic cholangiocarcinoma 22. Mechanistically, m7G tRNA modification discriminatively enhanced mRNA translation of genes in cell-cycle and epidermal growth factor receptor (EGFR) 22. Our results indicate *WDR4* as a hepatoblastoma predisposition gene. In the future, the role of METTL1 should be investigated in the tumorigenesis of hepatoblastoma.

Some limitations of this study should be discussed. First, several factors may increase hepatoblastoma risk, including defective development (e.g., maturation and low birth weight), harmful environmental exposures, and susceptibility genes. Here, we failed to include the former two factors in the study. Second, we did not find significant SNPs in the single-locus analysis. Many more potential functional SNPs in the *WDR4* gene should be interrogated. Third, the number of cases was relatively small, and our results should be interpreted carefully. Finally, biologically relevant evidence is lacking that *WDR4* has a role in hepatoblastoma pathogenesis due to the novelty of this gene. *In vitro* and *in vivo* functional analyses are warranted to explore the implication of WDR4 in hepatoblastoma tumorigenesis in the future direction.

Overall, we demonstrated that the presence of two more selected *WDR4* gene SNPs might increase the risk of hepatoblastoma in girls. The findings should be validated in large case-control studies.

## Supplementary Material

Supplementary figures and tables.

## Figures and Tables

**Table 1:** Association between *WDR4* gene polymorphisms and hepatoblastoma susceptibility

Genotype	Cases (N=308)	Controls (N=1444)	*P* ^a^	Crude OR (95% CI)	*P*	Adjusted OR (95% CI) ^b^	*P* ^b^
**rs2156315 C>T (HWE=0.063)**							
CC	181 (58.77)	858 (59.42)		1.00		1.00	
CT	109 (35.39)	526 (36.43)		0.98 (0.76-1.28)	0.894	0.99 (0.76-1.28)	0.908
TT	18 (5.84)	60 (4.16)		1.42 (0.82-2.47)	0.210	1.42 (0.82-2.46)	0.213
Additive			0.521	1.07 (0.87-1.32)	0.520	1.07 (0.87-1.32)	0.515
Dominant	127 (41.23)	586 (40.58)	0.833	1.03 (0.80-1.32)	0.832	1.03 (0.80-1.32)	0.822
Recessive	290 (94.16)	1384 (95.84)	0.192	1.43 (0.83-2.46)	0.194	1.43 (0.83-2.46)	0.198
**rs2156316 C>G (HWE=0.459)**							
CC	136 (44.16)	640 (44.32)		1.00		1.00	
CG	138 (44.81)	652 (45.15)		1.00 (0.77-1.29)	0.976	1.00 (0.77-1.30)	0.999
GG	34 (11.04)	152 (10.53)		1.05 (0.70-1.60)	0.809	1.05 (0.70-1.60)	0.808
Additive			0.870	1.02 (0.84-1.22)	0.870	1.02 (0.84-1.23)	0.857
Dominant	172 (55.84)	804 (55.68)	0.958	1.01 (0.79-1.29)	0.958	1.01 (0.79-1.30)	0.936
Recessive	274 (88.96)	1292 (89.47)	0.791	1.06 (0.71-1.56)	0.791	1.05 (0.71-1.56)	0.798
**rs6586250 C>T (HWE=0.897)**							
CC	248 (80.52)	1152 (79.78)		1.00		1.00	
CT	56 (18.18)	275 (19.04)		0.95 (0.69-1.30)	0.732	0.95 (0.69-1.30)	0.744
TT	4 (1.30)	17 (1.18)		1.09 (0.37-3.28)	0.874	1.08 (0.36-3.25)	0.888
Additive			0.822	0.97 (0.73-1.29)	0.823	0.97 (0.73-1.29)	0.826
Dominant	60 (19.48)	292 (20.22)	0.768	0.95 (0.70-1.30)	0.768	0.96 (0.70-1.30)	0.777
Recessive	304 (98.70)	1427 (98.82)	0.859	1.10 (0.37-3.31)	0.859	1.09 (0.37-3.27)	0.874
**rs15736 G>A (HWE=0.966)**							
GG	244 (79.22)	1146 (79.36)		1.00		1.00	
GA	60 (19.48)	281 (19.46)		1.00 (0.74-1.37)	0.986	1.01 (0.74-1.38)	0.964
AA	4 (1.30)	17 (1.18)		1.11 (0.37-3.31)	0.858	1.10 (0.37-3.29)	0.867
Additive			0.924	1.01 (0.77-1.34)	0.924	1.02 (0.77-1.34)	0.909
Dominant	64 (20.78)	298 (20.64)	0.955	1.01 (0.75-1.37)	0.955	1.01 (0.75-1.37)	0.936
Recessive	304 (98.70)	1427 (98.82)	0.859	1.10 (0.37-3.31)	0.859	1.10 (0.37-3.28)	0.869
**rs2248490 C>G (HWE=0.387)**							
CC	137 (44.48)	643 (44.53)		1.00		1.00	
CG	140 (45.45)	652 (45.15)		1.01 (0.78-1.31)	0.953	1.01 (0.78-1.31)	0.936
GG	31 (10.06)	149 (10.32)		0.98 (0.64-1.50)	0.914	0.98 (0.64-1.50)	0.914
Additive			0.960	1.00 (0.83-1.20)	0.960	1.00 (0.83-1.20)	0.970
Dominant	171 (55.52)	801 (55.47)	0.988	1.00 (0.78-1.28)	0.988	1.00 (0.78-1.29)	0.972
Recessive	277 (89.94)	1295 (89.68)	0.894	0.97 (0.65-1.46)	0.895	0.97 (0.65-1.46)	0.889
**Combined effect of risk genotypes^c^**							
0-1	282 (91.56)	1364 (94.46)		1.00		1.00	
2-5	26 (8.44)	80 (5.54)	0.053	1.57 (0.99-2.49)	0.054	1.57 (0.99-2.49)	0.056

OR, odds ratio; CI, confidence interval; HWE, Hardy-Weinberg equilibrium. ^a^ χ^2^ test for genotype distributions between hepatoblastoma patients and controls. ^b^ Adjusted for age and gender. ^c^ Risk genotypes were carriers with rs2156315 TT, rs2156316 GG, rs6586250 TT, rs15736 AA and rs2248490 CC/CG.

**Table 2:** Stratification analysis of risk genotypes with hepatoblastoma susceptibility

Variables	rs2156315 (cases/controls)		AOR (95% CI) ^a^	*P* ^a^	Risk genotypes (cases/controls)		AOR (95% CI) ^a^	*P* ^a^
	CC/CT	TT			0-1	2-5		
**Age, month**								
<17	154/608	10/33	1.21 (0.59-2.52)	0.603	149/600	15/41	1.50 (0.81-2.78)	0.202
≥17	136/776	8/27	1.67 (0.74-3.76)	0.214	133/764	11/39	1.61 (0.81-3.23)	0.177
**Gender**								
Females	116/567	10/28	1.75 (0.83-3.69)	0.145	111/561	15/34	2.23 (1.17-4.23)	0.014
Males	174/817	8/32	1.16 (0.53-2.57)	0.713	171/803	11/46	1.12 (0.57-2.20)	0.749
**Clinical stages**								
I+II	149/1384	10/60	1.53 (0.76-3.05)	0.231	146/1364	13/80	1.50 (0.82-2.77)	0.191
III+IV	83/1384	6/60	1.69 (0.71-4.03)	0.237	80/1364	9/80	1.93 (0.93-3.99)	0.076

AOR, adjusted odds ratio; CI, confidence interval. ^a^ Adjusted for age and gender, omitting the corresponding factor.
