# Supplementary material for: WDR4 gene polymorphisms increase hepatoblastoma susceptibility in girls
Source: J Cancer. 2022 Sep 21;13(12):3342–7. doi: 10.7150/jca.76255 (PMC9516011; doi:10.7150/jca.76255)
Supplement: Supplementary file 1 — Supplementary figures and tables. [file jcav13p3342s1.pdf]

Table S1. Frequency distribution of selected variables in hepatoblastoma patients and cancer-free controls

Variables	Cases (n=313)		Controls (n=1446)		P [a]
	No.	%	No.	%	
Age range, month	0.03-149.97		0.004-156.00		
Mean ± SD	23.75 ± 25.93		25.23 ± 19.38		0.251 [b]
<17	168	53.67	642	44.40	
≥17	145	46.33	804	55.60	
Gender					0.983
Female	129	41.21	595	41.15	
Male	184	58.79	851	58.85	
Clinical stages					
I	97	30.99			
II	63	20.13			
III	64	20.45			
IV	27	8.63			
NA	62	19.81			

SD, standard deviation, NA, not available.

[a] Two-sided χ^2 test for distributions between hepatoblastoma cases and cancer-free controls.

[b] T-test for age distribution between hepatoblastoma patients and cancer-free controls.
